# Autosomal dominant mesomandibular fibro-osseous dysplasia: a self-resolving inherited fibro-osseous lesion of the jaws

**DOI:** 10.3389/fphys.2012.00458

**Published:** 2012-12-06

**Authors:** Ioannis G. Koutlas, Cynthia L. Forsman, Stephanos Kyrkanides, William S. Oetting, Anna Petryk

**Affiliations:** ^1^Division of Oral and Maxillofacial Pathology, School of Dentistry, University of MinnesotaMinneapolis, MN, USA; ^2^Department of Genetics, Cell Biology and Development, University of MinnesotaMinneapolis, MN, USA; ^3^Department of Pediatrics, University of MinnesotaMinneapolis, MN, USA; ^4^Department of Orthodontics and Pediatric Dentistry, School of Dental Medicine, State University of New YorkStony Brook, NY, USA; ^5^Department of Experimental and Clinical Pharmacology, School of Pharmacy, University of MinnesotaMinneapolis, MN, USA

**Keywords:** inherited, benign fibro-osseous lesion, jaws, mandible, twisted gastrulation

## Abstract

A hereditary congenital condition characterized by a fibro-osseous lesion sharing some features with fibrous dysplasia and affecting the middle aspect of the mandible is presented. The condition was initially described as congenital monostotic fibrous dysplasia in two siblings, a male and a female. However, there is sufficient evidence that the disorder is autosomal dominant since it has been encountered in two of four children, both males, of the female propositus and one child, a boy, of the male propositus. All patients presented at birth or right after birth with enlargement of the middle part of the mandible. Radiographs from affected individuals have shown mesomandibular enlargement with irregular trabeculation akin of “ground-glass” appearance. Histologically, samples from all patients revealed woven bone proliferation in a cellular fibroblastic stroma. Interestingly, the originally described siblings, now in their 30s, have no evidence of jaw lesions either radiographically or clinically, thus indicating that the condition is self-limiting or self-resolving. An autosomal dominant mode of inheritance with apparent male predilection is favored. The molecular basis of this condition is currently unknown. However, the location of the lesions in the middle aspect of the mandible suggests dysregulation of Bone Morphogenetic Protein (BMP) signaling since BMPs regulate mandibular morphogenesis *in utero*, particularly in the medial region as well as postnatal bone remodeling. Immunohistochemical evaluation for a BMP-binding protein Twisted Gastrulation (TWSG1) revealed mosaic pattern of staining, with some cells, including osteoclasts, strongly stained and others exhibiting faint or no staining, thus supporting active regulation of BMP signaling within the lesion. Future investigations will determine if dysregulation of BMP signaling plays a causative role or rather reflects secondary activation of repair mechanisms and/or bone remodeling.

## Introduction

Benign fibro-osseous lesions (BFOLs) encompass a group of pathologic processes characterized by replacement of normal bone by generally cellular fibrous tissue and newly formed osseous, or so-called cemento-osseous, deposits. Included in the group of BFOL of the jaws are: (1) ossifying fibroma, a neoplasm, (2) fibrous dysplasia a condition associated with known mutations to the G protein subunit α (G_s_α), encoded by the *GNAS* gene at 20q13, that couples hormone receptors to intracellular cAMP, and (3) a variety of fibro-osseous processes referred to as (cemento)-osseous dysplasias subclassified by their clinicoradiographic characteristics. Familiarity with their clinical, radiographic, and in some cases histopathologic characteristics is important for management that can vary, depending on the type, from periodic observation, surgical recontouring for functional and esthetic purposes, to tumor resection.

Most cases are non-hereditary. However, the literature does contain cases of pedigrees with a certain type of BFOL. There are few reports of familial gigantiform cementoma (Cannon et al., 1980; Young et al., 1989; Oikarinen et al., 1991; Finical et al., 1999), florid osseous dysplasia (Musella and Slater, 1989; Coleman et al., 1996), periapical cemental dysplasia (Sedano et al., 1982; Thakkar et al., 1993), as well as alleged examples of familial fibrous dysplasia with craniofacial involvement (Talley, 1952; Reitzik and Lownie, 1975; Lemli, 1977; Alvarez-Arratia et al., 1983; Pierce et al., 1985; Sarkar et al., 1993; Hsissen et al., 1997). Also, a pedigree has been described in which one member featured “polyostotic fibrous dysplasia” while other members cherubism (Zohar et al., 1989). Fibro-osseous lesions of the jaws, referred to by some authors as cemento-ossifying fibromas (Jackson et al., 1990; Aldred et al., 2006), have been encountered in patients with hereditary hyperparathyroidism (Rosen and Palmer, 1981) caused by mutations in the tumor suppressor gene *HRPT2*.

In 1979, El Deeb et al. (1979) described, under the term congenital monostotic fibrous dysplasia, two siblings, a male and a female, presenting with a fibro-osseous process of the anterior middle aspect of the mandible exhibiting, clinicoradiographically and histopathologically, features suggestive of fibrous dysplasia. In 2006, the son of the female sibling presented with the same lesion. Extensive evaluation of the family was undertaken that resulted in the identification of affected offsprings of the two siblings. As it will be elucidated later in this report, this condition is unique, self-regressing, and disappearing with age. In our opinion, the term autosomal dominant mesomandibular fibro-osseous dysplasia (ADMFOD) best describes this condition. In addition, since misregulation of the Bone Morphogenetic Protein (BMP) pathway has been suggested to play a role in the pathogenesis of fibrous lesions in the bone (Kiss et al., 2010), we examined (by immunohistochemistry) if BMP binding protein Twisted Gastrulation (TWSG1) is present in either bone cells or fibrous tissue within the lesion in one of the affected members.

## Materials and methods

### Immunohistochemistry

Formalin-fixed paraffin-embedded tissue was sectioned at 5 μM and mounted on Superfrost Plus slides (Fisher Scientific, Pittsburgh, PA). Sections were deparaffinized in xylenes and rehydrated through a series of graded ethanol. Endogenous peroxidase activity was blocked by incubating sections in a 3% hydrogen peroxide/methanol solution for 10 min and then washed in PBS. Slides were placed in Antigen Unmasking Solution diluted to manufacturer's recommendation (Vector Laboratories, Burlington, CA) and microwaved for 20 min. Once cooled, slides were washed and then blocked in PBS containing 5% goat serum (Invitrogen, Carlsbad, CA) and 0.3% Triton X-100 (Fisher Scientific) for 60 min at room temperature. Sections were incubated with monoclonal anti-hTWSG1 antibody (2F3; Abnova, Taipei City, Taiwan) at 1:500 dilution overnight at 4°C. Detection was accomplished using SuperPicture Kit (DAB, Broad Spectrum, Life Technologies, Carlsbad, CA) following manufacturer's instructions.

## Background

### Description of the pedigree

**Figure 1:**
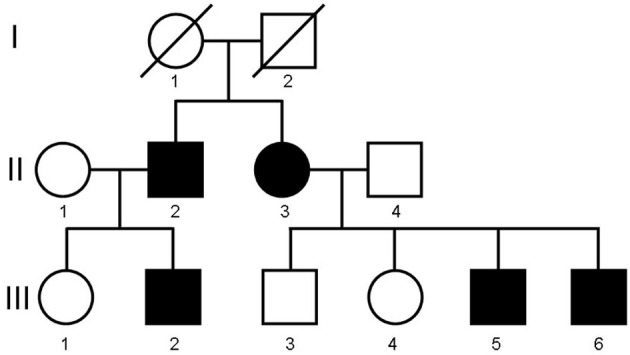
**Family pedigree**.

The description of the propositus and his sibling has been previously published (El Deeb et al., 1979). However, to thoroughly present the condition we deem it necessary to review the characteristics of the initially affected siblings in conjunction with the recently discovered affected members of the pedigree. The pedigree is depicted in Figure 1.

#### Patient II-2 (propositus)

**Figure 2:**
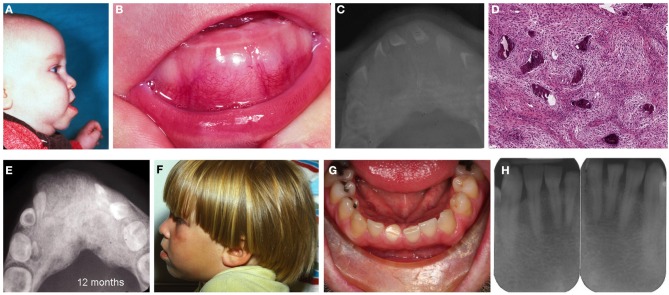
**Patient II-2. (A)** Profile of the patient exhibiting enlargement and protrusion of the mandible, **(B)** lobular enlargement of the mandible, **(C)** occlusal radiograph depicting enlargement of the middle aspect of the mandible characterized by a ground-glass pattern, **(D)** loose cellular fibroblastic proliferation with deposits of woven bone, **(E)** radiograph revealing irregular ground glass-like appearance of the mandible and splaying of teeth. **(F)** profile of the patient at age 3 year, **(G)** patient as an adult at age 33 years. No evidence of jaw enlargement was present, **(H)** no evidence of ground glass appearance or enlargement.

The male propositus presented at 3 months of age with progressing congenital enlargement of the anterior middle aspect of the mandible measuring 7.0 × 2.0 × 1.0 cm (Figures 2A,B). His medical history was non-contributory and he was the outcome of a normal pregnancy and delivery with no known family history of jaw lesions or consanguinity. Radiographically, the lesion was generally radiopaque with ill-defined borders and featured displacement and splaying of teeth and mimicking fibrous dysplasia (Figure 2C). Serum calcium, phosphorus and alkaline phosphatase levels were within normal limits. A biopsy revealed spindle cell proliferation of fibroblast-like cells with areas of early osteoid formation and deposits of irregular spicules of woven bone with limited osteoblastic rimming (Figure 2D). The rendered diagnosis at that time was BFOL, probable fibrous (fibro-osseous) dysplasia. Follow-up of the patient was recommended with no additional surgical procedures to be undertaken at that time.

Subsequent radiographs at age 10 and 12 months (Figure 2E) revealed progressive enlargement with distal and lingual expansion and increase in the density of the abnormal bone but without obvious facial abnormality. Progressive regression of the lesion was appreciated at 26 and 36 months (Figure 2F), which was attributed by the clinicians “to the normal growth of the patient,” meaning, most likely, that the lesion ceases to grow and becomes incorporated into the developing jaw. Throughout this period the patient did not reveal any evidence of café-au-lait spots or other skeletal abnormalities.

The male propositus, now at 33 years of age, does not present clinical (Figure 2G) or radiographic (Figure 2H) abnormalities of the mandible. Only arrested development with signs of attrition of the right permanent central mandibular incisor and focal diastema were appreciated. He has two children, an unaffected daughter (III-1) and an affected son (III-2). His son has not undergone any radiographic or surgical evaluation of his lesion.

#### Patient II-3

**Figure 3:**
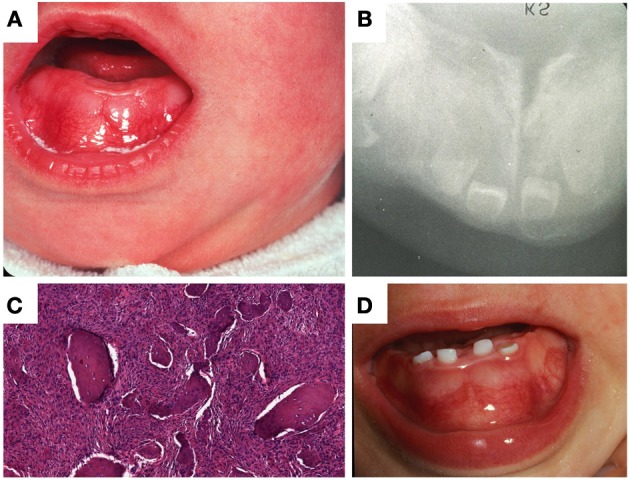
**Patient II-3. (A)** Lobular enlargement of the mandible, **(B)** ground glass appearance of bone and splaying of teeth, **(C)** brisk fibroblastic proliferation and irregular bone deposits with limited osteoblastic rimming, **(D)** at age 12 months there was enlargement of the mandible and splaying of teeth.

In 1978, the sibling of the propositus, a female infant, presented with congenital mesomandibular enlargement (Figure 3A). Radiographic (Figure 3B) and histopathologic (Figure 3C) features were essentially similar to her brother's. Follow-up at 12 months revealed persistent enlargement with splaying of teeth (Figure 3D) and signs of regression. Judging from the progressive reduction of both patients' lesions it was decided that no additional surgical treatment was required. After 1980 there was no recorded follow-up in the charts of both siblings.

#### Patient III-5

**Figure 4:**
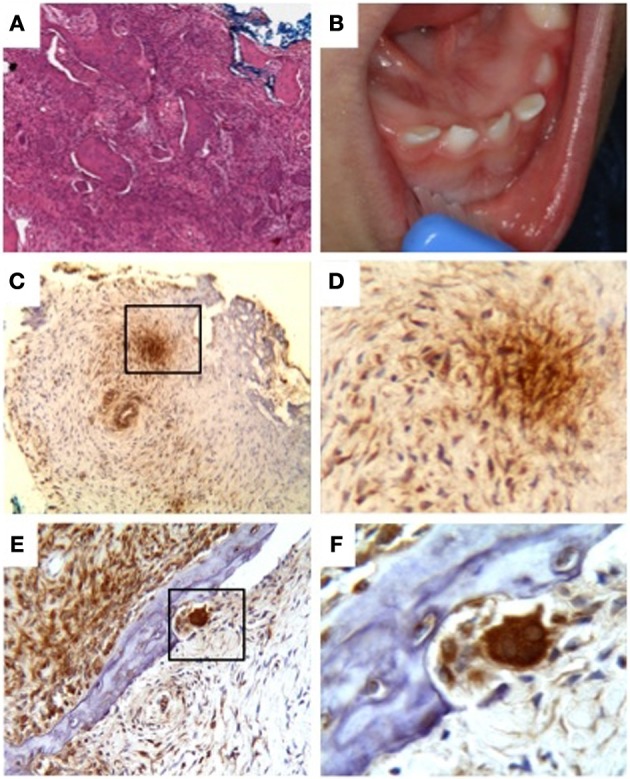
**Patient III-5. (A)** Irregular deposits of bone in a brisk fibroblastic stroma, **(B)** enlargement of the mandible with splaying of teeth, **(C–F)** immunodetection of TWSG1 in fibrous tissue, osteoclasts, and osteocytes within the lesion, **(C)** mosaic pattern throughout the sample with areas of high expression next to areas of low or no detectable expression, even within the same cell type, **(D)** 40x magnification of the boxed area in **(C)** showing primarily fibroblastic TWSG1 expression, **(E)** osteoclast in a resorption pit shows a high level of TWSG1 expression. TWSG1 expression is also detected in fibrous tissue adjacent to bony deposit, **(F)** 40x magnification of the boxed area in **(C)** showing strong TWSG1 expression in the osteoclast and expression in osteocytes.

In 2006, a 3-month-old male infant was examined by Ioannis G. Koutlas for progressive enlargement of the middle aspect of the mandible. Initially, relationship of the infant to any of the siblings described above was unknown to the examiner. Upon further discussion of the striking resemblance of the lesion to the reported patients by El Deeb et al. (1979) and the rarity of this congenital lesion, a direct familial relationship to the previous cases was established. Prior to being seen in the clinic, the infant had radiographic evaluation and incisional biopsy at a local hospital diagnosed as fibrous dysplasia. Histologic preparations revealed an essentially similar benign fibro-osseous process (Figure 4A).

Clinical evaluation of the patient's mother (patient II-3) did not reveal any clinical lesions. There was only transposition between the right mandibular canine and first premolar. There was no evidence of radiographic abnormality. A follow-up of the male infant was recommended. At 12 (Figure 4B) and 36 months the lesion was still present, albeit smaller. The decrease in size was apparently relative to the normal increase in size of the mandible. In addition, at 12 months, left sided limb enlargement was noticed (approximately 1 cm leg size discrepancy) as well as slight asymmetry between the right and lefts hands and right and left feet. The patient underwent a complete skeletal survey at age 13 months. Besides the mandibular lesion there were no other osseous abnormalities present to suggest a similar dysplastic process in other bones. The left femur and tibia were minimally larger than the right but with no intrinsic bone abnormality. Follow up radiographic evaluation of the chest, pelvis, hands and feet including bone length studies have been undertaken from 2007 to 2011. The last bone length study confirmed the right lower extremity measuring 54.4 cm from the superior femoral head to the tibial plafond while the left lower extremity measured 55.3 cm. The left femur was 0.5 cm longer than the right, and the left tibia also 0.5 cm longer than the right. During this period of 5 years, bilateral coxa valga deformities were also appreciated; however, they showed improvement with time. Chest evaluation did not reveal any abnormalities of osseous structures. Since other affected members did not report or reveal, after clinical evaluation, such clinical limb anomalies, these findings are most likely unrelated to the jaw lesion.

In order to examine if BMP binding protein TWSG1 was present in either bone cells or fibrous tissue within the lesion, sections of lesional tissue obtained from this patient were evaluated by immunohistochemistry with monoclonal antibody against human TWSG1. Immunoreactivity was observed in a mosaic pattern within the lesion with some groups of cells stained strongly and other groups exhibiting low to no staining (Figures 4C–F). The staining was cytoplasmic and detected in fibroblasts, osteocytes and the strongest staining was seen in osteoclasts.

#### Patient III-6

**Figure 5:**
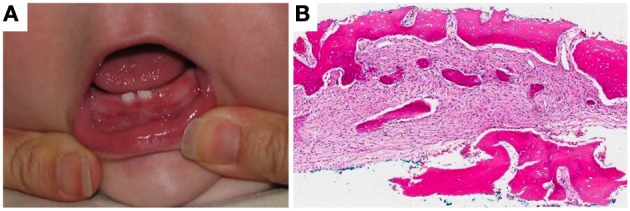
**Patient III-6. (A)** Subtle enlargement of the mandible and **(B)** loose fibroblastic proliferation with woven bone deposits. Very limited osteoblastic rimming was noted.

In 2009, another affected male infant was born to the female patient II-3 with mesomandibular enlargement, however, not as noticeable as his brother's (Figure 5A). The lesion was also biopsied exhibiting a similar benign fibro-osseous process (Figure 5B). The family has two older unaffected children.

## Discussion

Inherited fibro-osseous lesions of the jaws are rare and appear in the literature as examples of gigantiform cementomas (Cannon et al., 1980; Young et al., 1989; Oikarinen et al., 1991; Finical et al., 1999), familial florid osseous dysplasia (Musella and Slater, 1989; Coleman et al., 1996), and periapical cemental dysplasias (Sedano et al., 1982; Thakkar et al., 1993). The term gigantiform cementoma has been used in the past as a synonym for florid osseous dysplasia and examples of familial gigantiform cementomas were grouped with familial cases of florid osseous dysplasia (Coleman et al., 1996). Also, prior to establishment of cemento-osseous lesions as a separate entity, they have been published as examples of familial fibrous dysplasia (Talley, 1952). Gigantiform cementomas and florid osseous dysplasia tend to follow the cycle of fibrous dysplasia and they cease to grow around the fifth decade of life. Currently, the term gigantiform cementoma is restricted to certain sporadic or familial cases presenting as multifocal rapid and expansive enlargements of both the maxilla and mandible leading to facial deformity, which can be occasionally severe (Cannon et al., 1980; Young et al., 1989; Finical et al., 1999).

A most unique case of hereditary fibro-osseous lesion was described by Chatterjee and Mazumder (1967) originating in the mucoperiosteum of the middle aspect of both the maxilla and the mandible, affecting the alveolus and extending to the palate causing substantial disfigurement. It has been referred to as “hippopotamus face” (Kundu and Pan, 1979), an unfortunate characterization.

Very little is known about the pathogenesis of florid osseous dysplasia and gigantiform cementoma, sporadic, or familial. We were able to identify a single reference, presented as an abstract, of *GNAS* mutation in two examples of florid cemento-osseous dysplasia and one case of cemento-ossifying fibroma (Al-Adnani et al., 2005). The validity of these results has not been confirmed in other studies that failed to show *GNAS* mutations in cases of ossifying (cementifying) fibromas and cemento-osseous dysplasias (Toyosawa et al., 2007; Patel et al., 2010).

Prior to the identification of the gene associated with McCune-Albright syndrome and cases of polyostotic and monostotic fibrous dysplasia, Dr. Happle postulated that certain sporadic syndromes that display a mosaic pattern of involvement of skin and organs (McCune-Albright syndrome, Schimmelpenning-Feuerstein-Mims syndrome, Proteus syndrome) are the result of an autosomal “dominant” lethal gene leading to loss of the zygote *in utero* (Happle, 1987). In order to survive, mutated cells must be intermingled with normal cells creating a mosaic. This mosaic can result from either an early somatic or a gametic half chromatid mutation. Now it is known that fibrous dysplasia, monostotic, polyostotic, or as part of the McCune-Albright syndrome is caused by postzygotic (somatic) mutations in the gene encoding the alpha subunit of the stimulatory G protein within the GNAS complex locus (Weinstein et al., 1991; Riminucci et al., 2006). Depending on the timing of such mutations, patients will have widespread or limited manifestations. It has been also shown that individual lesions of fibrous dysplasia are themselves a mosaic of mutated and normal cells (Bianco et al., 1998). With time, mutated cells population is decreased with normal cells predominating thus leading to arrested growth of lesions. A recent study comparing bone tissue samples from patients with fibrous dysplasia to patients without it, besides confirming the involvement of G protein coupled pathway, implicated also BMP signaling pathway in the pathogenesis of these lesions (Kiss et al., 2010). Fibrous dysplasia and non-fibrous dysplasia subjects could be clearly separated by differences in the expression of BMP2, BMP4, BMPR1A, BMPR2, and other members of the BMP pathway. It has been postulated that since mutant cells in fibrous dysplasia are clonal fibroblast-like osteoprogenitor cells demonstrating high expression of the c-fos proto-oncogene, fibrous dysplasia may be after all a neoplasm (Cohen, 2001).

Although it is well established that the mutations causing fibrous dyplasia occur postzygotically, thus excluding the possibility of the disease to be inherited, there are reported cases of familial fibro-osseous lesions, clinically and histologically, similar or even indistinguishable from fibrous dysplasia (Reitzik and Lownie, 1975; Lemli, 1977; Alvarez-Arratia et al., 1983; Pierce et al., 1985; Sarkar et al., 1993). They are presumably either the result of gametic half chromatid mutation or hereditary conditions caused by a presently unknown gene mutation that leads to a phenotype similar to fibrous dysplasia. Linkage studies in these families are necessary to confirm the proposed diagnoses of familial fibrous dysplasia. The only pedigree among those reported as alleged examples of familial fibrous dysplasia with jaw manifestation is the family presented by Pierce et al. (Pierce et al., 1985). The investigators did not succeed in finding a mutation in *GNAS* or in the gene causing cherubism (Mangion et al., 2000). Based on the published clinical, radiographic, and histologic figures it appears that the family described by Pierce et al. (Pierce et al., 1985, 1996), represents either familial florid osseous dysplasia or a unique entity certainly different from fibrous dysplasia. Finally, the family described by Zohar et al. (Zohar et al., 1989) as presenting with a combination of fibrous dysplasia and cherubism has just familial cherubism.

The condition affecting the family described herein was previously referred to in the literature as monostotic fibrous dysplasia (El Deeb et al., 1979). Based on the above argument against the hereditary nature of fibrous dysplasia and the observations that a) it affected the mandibular midline, hardly a preferred site for fibrous dysplasia, as well as b) its clinical behavior characterized by not only a self-limiting process but a self resolving one, we decided to use the term fibro-osseous dysplasia. We elected not to refer to the process as osteofibrous dysplasia, to distinguish it from the familial form of osteofibrous dysplasia (Hunter and Jarvis, 2002; Karol et al., 2005).

Osteofibrous dysplasia, known also as “osteitis fibroma,” a “variant of fibrous dysplasia,” and “ossifying fibroma of long bones,” is a benign bone dysplastic process affecting primarily the cortex of the tibia and/or the fibula of skeletally immature patients (Park et al., 1993). Patients often present with anterolateral bowing of the affected bones that often lead to fractures (Karol et al., 2005). Rarely, lesions disappear spontaneously (Campanacci and Laus, 1981). Histologically, osteofibrous dysplasia is characterized by fibroblastic proliferation associated with woven bone rimmed by osteoblasts. Because of the presence of osteofibrous dysplasia-like areas in adamantinomas a close relationship of the two lesions has been suggested, with osteofibrous dysplasia being a precursor to or a regressive form of adamantinoma. No adamantinoma has occurred in patients with the rare familial form of osteofibrous dysplasia (Karol et al., 2005). Also, cases of osteofibrous dysplasia have not shown any *GNAS* mutation (Sakamoto et al., 2000).

The molecular mechanism associated with the familial fibro-osseous condition presented in this paper is unknown. However, the location of the lesions in the middle aspect of the mandible suggests dysregulation of BMP signaling. BMPs regulate mandibular morphogenesis, particularly in the medial region (Ferguson et al., 2000; Mina et al., 2002; MacKenzie et al., 2009). Moreover, differential expression of genes in the BMP pathway has been reported to distinguish patients with and without fibrous dysplasia (Kiss et al., 2010), and activation of BMP pathway due to mutations in BMP type 1 receptor has been linked to fibrodysplasia ossificans progressiva (Shore et al., 2006; Fukuda et al., 2009). BMPs regulate a number of cellular processes, including proliferation, differentiation, migration, apoptosis, and epithelial-mesenchymal interactions (Hallahan et al., 2003; von der Hardt et al., 2007; Wagner et al., 2010). Postnatally, BMPs play a role in regulating tissue homeostasis in physiological and pathological conditions, such as tissue regeneration (Simic and Vukicevic, 2007; Larman et al., 2009), bone remodeling (Pham et al., 2011), immune function (Passa et al., 2011; Tsalavos et al., 2011), and cancer (Thawani et al., 2010; Buijs et al., 2012).

BMP signaling is modulated in the extracellular space by BMP-binding proteins Noggin, Chordin, Chordin-like 1, TWSG1, and others (Ross et al., 2001; Scott et al., 2001; Balemans and Van Hul, 2002; Larman et al., 2009). In this paper, we limited the evaluation to the expression of TWSG1 as an example of a BMP-binding protein because of its known role in regulating BMP activity during mandibular morphogenesis (MacKenzie et al., 2009) and postnatal bone remodeling (Sotillo Rodriguez et al., 2009), both of which are relevant to the described pathology, as well as previously validated detection methods (Sun et al., 2010; Johnston et al., 2012). Expression of TWSG1 in the fibrous and osseous elements of the lesion suggests a role for BMP regulation. A high level of expression of TWSG1 in osteoclasts that are present in the resorption pits is consistent with its role in regulating BMP signaling in osteoclastogenesis (Sotillo Rodriguez et al., 2009). It also points to a possible role of active bone remodeling or other reparative mechanisms in limiting the extent of the lesion. Both BMPs and TWSG1 have been implicated in tissue regeneration (Simic and Vukicevic, 2007; Larman et al., 2009). It remains to be determined if other components of the BMP pathway are expressed and whether dysregulation of BMP signaling plays a causative role or rather reflects secondary activation in response to tissue damage. It is conceivable that localized upregulation of BMP antagonists may limit inappropriate activation of BMP signaling in the medial region of the mandible, contributing to the resolution of the lesions. Mutational analysis of genes in BMP pathway, including those encoding BMP ligands, BMP-binding proteins, BMP receptors, and downstream targets *Msx1* and *Msx2* (Vainio et al., 1993), may provide insight into the pathogenesis of this disease.

In summary, we present additional information on the clinicopathologic features of the family initially reported by El Deeb et al. (El Deeb et al., 1979) and we argue against the characterization of it as a familial form of monostotic fibrous dysplasia. We propose the term ADMFOD. It is a self-resolving unique fibro-osseous process among BFOL of the jaws.

### Conflict of interest statement

The authors declare that the research was conducted in the absence of any commercial or financial relationships that could be construed as a potential conflict of interest.
